# Structural quality of labor and delivery care in government hospitals of Ethiopia: a descriptive analysis

**DOI:** 10.1186/s12884-022-04850-5

**Published:** 2022-06-28

**Authors:** Negalign B. Bayou, Liz Grant, Simon C. Riley, Elizabeth H. Bradley

**Affiliations:** 1 Department of Health Policy and Management, Institute of Health, Jimma University, Jimma, Ethiopia; 2 Center for Population Health Sciences, Global Health Academy, Usher Institute of Population Health Sciences and Informatics, Scotland, University of Edinburgh, Scotland Edinburgh, United Kingdom; 3 Centre for Reproductive Health, University of Edinburgh, Scotland Edinburgh, United Kingdom; 4 Vassar College, Poughkeepsie, New York USA

**Keywords:** Quality, Structure, Labor, Delivery, Hospital, SNNPR, Ethiopia

## Abstract

**Background:**

Ethiopia has low skilled birth attendance rates coupled with low quality of care within health facilities contributing to one of the highest maternal mortality rates in Sub-Saharan Africa, at 412 deaths per 100,000 live births. There is lack of evidence on the readiness of health facilities to deliver quality labor and delivery (L&D) care. This paper describes the structural quality of routine L&D care in government hospitals of Ethiopia.

**Methods:**

A facility-based cross-sectional study design, involving census of all government hospitals in Southern Nations Nationalities and People’s Region (SNNPR) (*N* = 20) was conducted in November 2016 through facility audit using a structured checklist. Data collectors verified the availability and functioning of the required items through observation and interview with the heads of labor and delivery case team. An overall mean score of structural quality was calculated considering domain scores such as general infrastructure, human resource and essential drugs, supplies, equipment and laboratory services. Summary statistics such as proportion, mean and standard deviation were computed to describe the degree of adherence of the hospitals to the standards related to structural quality of routine labor and delivery care.

**Results:**

One third of hospitals had low readiness to provide quality routine L&D care, with only two approaching near fulfilment of all the standards. Hospitals had fulfilled 68.2% of the standards for the structural aspects of quality of L&D care. Of the facility audit criteria, the availability of essential equipment and supplies for infection prevention scored the highest (88.8%), followed by safety, comfort and woman friendliness of the environment (76.4%). Availability skilled health professionals and quality management practices scored 72.5% each, while availability of the required items of general infrastructure was 64.6%. The two critical domains with the lowest score were availability of essential drugs, supplies and equipment (52.2%); and laboratory services and safe blood supply (50%).

**Conclusion:**

Substantial capacity gaps were observed in the hospitals challenging the provision of quality routine L&D care services, with only two thirds of required resources available. The largest gaps were in laboratory services and safe blood, and essential drugs, supplies and equipment. The results suggest the need to ensure that all public hospitals in SNNPR meet the required structure to enable the provision of quality routine L&D care with emphases on the identified gaps.

## Background

In 2015, 303,000 mothers died worldwide [1], 99% of these deaths occurred in low-and middle-income countries [2] with over two thirds in Sub-Saharan Africa (SSA). Ethiopia has one of the highest maternal mortality rates in SSA, with 412 deaths per 100,000 live births. The lifetime risk of a maternal death in Ethiopia is one in 48 women in 2015 [3], much higher than the average for SSA which is one in 36 [4].

Access to facilities that are adequately equipped and staffed with skilled birth attendants is a prerequisite for achieving a meaningful reduction in maternal mortality to ensure that all women get skilled maternity care [5]. Recent evidence shows that half of all maternal and one million newborn deaths can be prevented by providing high-quality care before, during and after childbirth. In low income countries, however, mothers receive less than half of the recommended practices in a typical maternity care visit [6]. Very low rates of skilled birth attendance, at 28% [3], coupled with low quality of care within health facilities is a major contributor to the high maternal mortality rate in Ethiopia [7].

Quality of health care involves proper performance of standardised interventions that are known to be safe, affordable and that have the ability to improve health outcomes and meet or exceed client expectations [8]. The most commonly used framework for assessing quality of healthcare is Donabedian’s structure-processes-outcomes conceptualization of quality [9]. Structural measures of healthcare quality are concerned with the descriptive characteristics of the physical state of facilities or providers that affect effectiveness of services including, buildings, equipment, staff, beds and others required as per the set standards [10, 11]. The assumption behind measuring structure is that the setting can be a strong determinant of care quality and given the proper system, good care will follow [10].

Interlinked resource constraints are major factors affecting the quality of Labor and Delivery (L&D) care provision [12]. The healthcare system should therefore ensure that women are cared for in the right place, at the right time, by the right people with the right skills to deliver high quality care [13]. Poorly prepared facilities can demotivate health workers [14], and can also negatively affect women’s perceptions of the quality service [15], ultimately weakening the link between the health facilities and community [16].

Productivity, competence, availability and responsiveness of skilled birth attendants are at the heart of good quality obstetric care services [12, 17, 18]. Both shortages [12] and an inadequate mix of expertise in a cohort of skilled attendants [19] are major obstacles in low income countries. This can affect users' perceptions of quality of care, further leading to low skilled birth attendance rates [20]. For instance, skilled attendants in Benin, Ecuador, Jamaica and Rwanda scored only 50% in the required skills, partly due to inadequate training [12]. Likewise, poor competence of skilled attendants contributed to poor quality of obstetric care in Ethiopia, despite advances in their number with a ratio of 14.2 skilled attendants for 100 expected deliveries [21].

The availability of essential equipment, drugs and supplies required for providing high quality obstetric care varies across low-income settings. Most of the equipment required to organize emergency obstetric care (EmOC) services are available in all health facilities in Bangladesh [22] and Afghanistan [23]. The proportion of basic infrastructure components at district hospitals is significantly lower than at provincial or regional and specialized hospitals in Afghanistan [23]. All health facilities in Burkina Faso, Ghana and Tanzania lacked essential equipment [24], and facilities in Bangladesh had inadequate supply of drugs and blood for transfusion [22].

In Ethiopia inadequate equipment of hospitals for treating common causes of maternal and perinatal mortality has been identified as a health system factor contributing to the tragedy [25]. Thermometers, sphygmomanometer, fetoscope, delivery sets, blank partographs, sterilizers, refrigerators, toilet facilities, and a clean water supply are commonly missing or nonfunctional in health facilities in North Gondar [26].

The capacity of health facilities to deliver high quality care is often a neglected topic despite its critical role in determining the behavior of the personnel in the system and thus, the quality of care offered and enjoyed [27]. Furthermore, weak organization and management of existing services can be the main reason for poor facility operation, which often needs minimal or no further spending on infrastructure or other improvement [28]. Data on certain facility inputs can be obtained from administrative records at national or sub-national level, but such data lack richness and detail for which a survey approach is often required [29]. Data on structural measures of quality of care is also captured through periodic nationwide surveys of Service Availability and Readiness (SARA) [30–32]. However, they do not generate detailed information as they are limited to tracer items of selected priority services. Therefore, towards closing this gap, this paper describes the structural quality of routine L&D care based on a facility audit of 20 government hospitals in Southern Nations Nationalities and People’s Region (SNNPR), Ethiopia. Specifically, it presents the degree to which the hospitals fulfilled the checklist of resources required to enable provision of quality routine L&D care per the standards of Ethiopian Hospital Alliance for Quality (EHAQ) [33].

## Methods

### Study setting and period

The study was conducted in SNNPR, which is located in the Southern and South-western parts of Ethiopia. It is the third populous region hosting about 20% of the country's population [34]. As of 2014, there were 20 government hospitals operating in the region [35]. The study was conducted in November 2016.

### Study design and sampling

This study employed a facility-based cross-sectional study design, involving census of all government hospitals in SNNPR (*N* = 20). The reason for focusing on government was that the majority (85%) of the formal sector health facilities in Ethiopia are managed by the government [4]. The indicators of structure quality that most directly affect quality outcomes identified by a previous study on quality of family planning [36], are consistent with the country’s indicators for L&D service at public hospitals [33, 37]. Facility readiness, i.e., the capacity to provide needed services, can only be validly measured through facility audit [37].

### Data collection tool and procedure

Data were collected using a structured checklist based on the country’s standards for heath facility quality [33]. The checklist had six sections to obtain the required data as comprehensively as possible, focusing on the general background of the hospital; availability of human resource by category and training status; availability of essential drugs, supplies and equipment in L&D ward; availability of laboratory services and safe blood; and availability of essential guidelines for key L&D care processes.

Availability of supportive organizational structures with feedback systems for Maternal and Neonatal Health Care (MNHC) Quality Assurance (QA) and Quality Improvement (QI) was also assessed, including for maternal and neonatal mortality audit function. Considering these as structural measures of quality, rather than process measures, is supported by several evidence including the World Health Organization (WHO) standards for improving quality of MNHC [38]. Maternal death audit reviews and waste management practices were similarly considered as structural attributes of quality of maternal health services in Tanzania [39]. The Ethiopia SARA also captures components of such support systems along with facility infrastructure and measures of service delivery environment [31, 32]. Maternal health service statistics were also reviewed on selected key performance indicators for the year 2015 to characterize the study hospitals. These included the expected number of births and attended births (both live and stillbirths), caesarean deliveries and institutional maternal deaths.

Data were collected by professional midwives who were trained for two days on the EHAQ change packages for L&D care and related guidelines, and data collection checklist. They verified the availability and functioning of each item through observation and by asking the head of the L&D case team, as appropriate. Each single variable on the checklist received a score of ‘1’ if the item was available and in working condition, or a score of ‘0’ otherwise. Room temperature was assessed by adapting a similar indicator from the WHO standards for improving quality of MNHC [38] to suit to the MNHC guide for hospitals in Ethiopia [40]. This was done by asking whether the hospitals had a mechanism to maintain a documented room temperature in the L&D ward between 20 and 30C^0^, with verification from records where possible. The process was closely supervised by experienced supervisors who were also trained for two days.

### Data analysis

An overall facility readiness score for a specific service is the cumulative availability of components required in a facility to deliver the service. It is an unweighted average of the number of items present and functioning, expressed as a percentage of the total number of items in that service [37]. Thus, the initial score of ‘1’ was converted into ‘100’ to create a 0–100 scale, so that an item of structural quality was assigned a value of 100 if it was present and functioning and 0 otherwise. Summary statistics such as proportion, mean and standard deviation were computed to describe the degree of adherence of the hospitals to the standards related to structural quality of routine L&D care [33]. We used box and whiskers plot to examine the dispersion of median percentage scores for the structural attributes across hospitals. Ethics review and approval was obtained from the Scientific and Ethics Review Boards of Jimma University of Ethiopia, and the University of Edinburgh, UK.

## Results

### Characteristics of the study hospitals

Seven (35%) of the hospitals were teaching hospitals. Half of the hospitals were general hospitals by level, 7(35%) were primary, and the rest, 3(15%), were referral hospitals. Almost all (99.1%) of the expected births were attended in the hospitals in 2015. Half of the hospitals reported to have attended births beyond the number they expected. One in five of the attended deliveries (20.3%) were caesarean deliveries with intact uterus. A total of 144 institutional maternal deaths were reported by all hospitals in the reference year (Table 1).

**Table 1:** Maternal health service statistics of public hospitals in SNNPR, Ethiopia, 2016

Hospital	Expected births	Attended births (live & stillbirths)		^a^Caesarean deliveries		Maternal deaths in the hospital
	**№**	**№**	**%**	**№**	**%**	**№**
Adare	3286	3799	115.6	242	6.4	2
Arba Minch	6895	3208	46.5	701	21.9	8
Bona	1227	1129	92.0	179	15.9	7
Bonga	1408	1732	123.0	195	11.3	2
Buta Jira	4284	3689	86.1	510	13.8	2
Chencha	931	755	81.1	98	13.0	0
Dilla	3600	3909	108.6	880	22.5	11
Durame	2052	2222	108.3	598	26.9	3
Gidole	1400	1286	91.9	140	10.9	0
Halaba	1482	2709	182.8	223	8.2	1
Hawassa	3955	4720	119.3	2020	42.8	38
Hossana	4395	4677	106.4	1144	24.5	6
Jinka	1800	2280	126.7	335	14.7	11
Karat	920	812	88.3	140	17.2	0
Leku	1252	1457	116.4	292	20.0	0
Mizan Aman	2004	2100	104.8	194	9.2	20
Sawula	1200	1206	100.5	270	22.4	3
Tercha	1000	942	94.2	251	26.6	4
Wolaita Soddo	3409	3511	103.0	921	26.2	24
Yirgalem	3032	2934	96.8	654	22.3	2
Total	**49,532**	**49,077**	**99.1**	**9987**	**20.3**	**144**

^a^ Proportion of caesarean deliveries is computed from total number of attended deliveries

### General infrastructure

Within the domain of general infrastructure, 14 hospitals (70%) had continuous electricity and water supply 24 h a day and 7 days a week. A total of 18 hospitals (90%) had a reliable backup source in case of power cut (e.g., an automatic generator, or one that can be started within 5 min). While 8 hospitals (40%) had telephone service for internal communication, only 1 hospital (5%) had the same service in the compound for use by patients or their families. Though 17 hospitals (85%) reported that they had suggestion box or log-book for handling compliant in the labor ward, only 9 (52.9%) regularly evaluated and documented the suggestions.

Eighteen hospitals (90%) had a functional ambulance or other vehicle stationed at the hospital for emergency transportation for clients. Laboring mothers go directly to labor ward before any administrative procedure, and emergency triage exists for sick pregnant mothers who are not in labor in 17(85%) and 13(65%) of the hospitals, respectively. In aggregate, the mean percentage score for availability of general infrastructure was found to be 64.6%. That is, 64.6% of the required items of general infrastructure were available on average.

### Human resource

A total of 440 fulltime skilled birth attendants of all categories (i.e., Gynecology and Obstetrics (Gyn/Ob) specialists, Integrated Emergency Surgical Officers (IESOs), General Practitioners (GPs), Health Officers (HOs), Nurses and Midwives) were working in maternity ward in all hospitals. The analysis by category showed that there was no fulltime Gyn/Ob specialist, GP and nurse in 8(40%), 10(50%) and 8(40%) of the hospitals, respectively. There was no hospital without a fulltime midwife, and 19(95%) of the hospitals had four or more fulltime midwives. All hospitals reported that one or more of the skilled attendants was always present at the hospital or on-call, 24 h a day and 7 days a week for routine L&D care including weekends. However, the available skilled attendants in only 9 hospitals (45%) had received refresher training on obstetric care during 12 months prior to the study. Taking these two indicators in to account, i.e., continuous availability of at least one of the skilled attendants at the hospital or on-call, and receipt of refresher training, the mean percentage score for availability of skilled birth attendants was 72.5%.

### Drugs, supplies and equipment

**Fig. 1:**
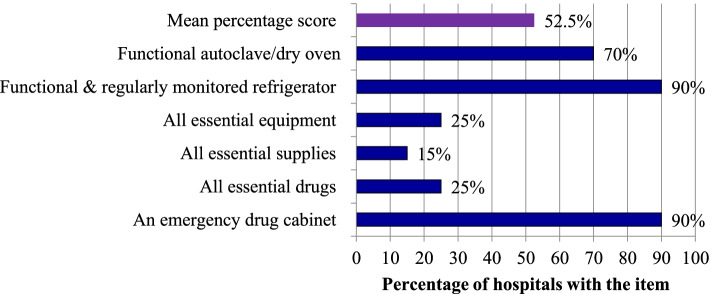
Availability of essential drugs, supplies and equipment in the L&D ward at public hospital in SNNPR, Ethiopia, 2016**. *Notes***: The following essential drugs, supplies and equipment should be available in the emergency drug cabinet of L&D ward. Drugs: Oxytocin injection, Misoprostol PO, Ergometrine, Nifedipine, Hydralazine, MgSO4, Diazepam, Lidocaine, Atropine, Calcium gluconate, Vit K, TTC eye ointment, Ampicillin IV, TDF/3TC/EFV (ARV drugs), Nevirapine syrup, Aminophylline, Adrenaline, and Hydrocortisone. Supplies: HIV test kits, 40% glucose, IV fluids (crystalloids), IV Cannula, Syringe & needle, Sterile gloves, IV stand, Mask for oxygen administration, Towels for drying and wrapping new-born babies, and Long sleeve glove for removal of retained placenta. Equipment: Sphygmomanometer, Stethoscope, Portable suction machine, Pinnard stethoscope (fetoscope)/doppler, Ultra sound, Thermometer, Filled oxygen tank with flow meter, Nasal prongs for oxygen administration, Catheter for oxygen administration, 5 delivery sets (at least 2 sterile), Sterile suture kit, Forceps, Vacuum extractor, Urinary catheter, Stand lamp, Speculum for vaginal examination, Craniotomy set, Sterilizer (steam or dry), a new born sized ambubag (with volume of 250 ml/less), Bed with accessories, Cord cutting/clumping set, Radiant warmer, Weighing scale for baby, Tape to measure baby length and head circumference, Functioning clock, 2 episiotomy sets, Suction bulb for new-born resuscitation, Emergency drug cabinet, and a functional refrigerator

On the day of assessment, 18 hospitals (90%) had an emergency drug cabinet in the maternity ward, and only 5(25%) of the hospitals had all essential drugs required for L&D care service provision. All hospitals had injectable oxytocin. However, magnesium sulphate, TTC eye ointment and anti-retroviral (ARV) drugs for HIV positive mothers were each missing in two hospitals. Similarly, nevirapine syrup was missing in one hospital for neonates exposed to HIV. The same was true with availability of essential equipment where five hospitals (25%) had all essential equipment. A filled oxygen tank with flow meter, ultrasound, and a bed with accessories were frequently absent. They were available in L&D ward in 17(85%), 16(80%) and 13(65%) of the hospitals, respectively. In addition, 10% of the hospitals lacked a functional and regularly monitored refrigerator, while 30% lacked a functional autoclave or dry oven in the ward. The availability of supplies was much lower. All essential supplies were available in only three hospitals (15%). Importantly, availability of towels for drying and wrapping new-born babies was 60%, and two hospitals (10%) did not have HIV test kits (i.e., KHB or Stat pack)*.* On average, 52.5% of the required essential drugs, supplies and equipment were available in the L&D ward of the study hospitals (Fig. 1).

### Infection prevention (IP) equipment and supplies

Personal protective equipment such as, goggle, boots, apron and gown were available in the L&D ward for IP in 65% of the hospitals. Consumables such as alcohol, chlorine, detergents, gloves and syringes were available in 95% of the hospitals for the same purpose. All hospitals had adequate safety boxes, while 95% had adequate yellow and black color-coded waste collecting bins for waste segregation. The mean score for availability of personal protective equipment and consumables was 88.8%.

### L&D environment

When the L&D environment was assessed whether it was woman-friendly, almost all (95%) of the hospitals had well ventilated rooms for women in both the first and second stage of labor. Appropriate room temperature (i.e., 20-30C^0^) was maintained in 70% of the hospitals. Moreover, only 65% of the hospitals had sufficient space for women in the first stage labor to walk around, and to have one companion. Most hospitals (80%) had tap water and functional sinks with detergents in maternity ward. A functional and clean toilet with door, and hand washing basin with soap was available in only 60% of the hospitals. Overall, 76.4% of the required safety, comfort and privacy standards were met by the hospitals (Table 2).

**Table 2:** Availability of resources required to ensure safe, comfortable and woman friendly L&D environment at public hospitals in SNNPR, Ethiopia, 2016

Items	Hospitals with item	
	**№**	**%**
Well ventilated rooms for first and second stage labor	19	95
Room temperature for first and second stage labor maintained at 20^0^C-30C^0^	14	70
Screens or curtains to ensure privacy	17	85
Sufficient space for women at first stage to walk around & for one companion	13	65
Hand washing and toilet facilities:		
•*Tap water*	16	80
•*Functional sinks with detergents*	16	80
•*Functional & clean toilet with door, & hand washing basin with soap*	12	60
Mean percentage score	76.4	

### Laboratory service

All the lab tests required for a mother in labor were available 24 h a day and 7 days a week in only 15% of the hospitals. Specifically, tests for Cerebrospinal Fluid (CSF) microscopy, liver function and renal function were not always available in 40%, 35% and 35% of the hospitals, respectively. Hospitals did not always provide serum protein and albumin tests (25%), CD4 count or HIV plasma viral load tests (20%), and HIV tests (10%) for women in need. On the other hand, 85% of the hospitals reported to have had adequate blood supply from a blood bank, which was stored properly in a fridge, with a temperature record. All of these hospitals provided blood without replacement. Only 50% of the required standards for availability of laboratory services and supply of safe blood were met in this study.

### Quality assurance

All essential guidelines and protocols were available in the L&D ward in only half of the hospitals. Particularly, the management protocol on selected obstetrics topics, from the Federal Ministry of Health (FMoH) of Ethiopia (2010 version), and infection prevention guidelines were available in 75% and 85% of the hospitals, respectively. In regard to QI activities, 13(65%) of the hospitals did establish a MNHC QI committee from different case teams and assigned a focal person to coordinate the committee. However, documented regular meetings (at least every two weeks) were achieved only by 9(69.2%) of these hospitals. Similarly, 75% of the hospitals had been conducting monthly maternal and neonatal death rounds or audits and were providing recommendations. Of these, 14(93.3%), reported that they were actually implementing the recommendations. Overall, 72.5% of the standards for QA practices were met.

In general, the hospitals had fulfilled 68.2% of the standards for the structural aspects of quality of L&D care. Availability of essential equipment and supplies for infection prevention was the category that scored the highest (88.8%). In contrast, the two critical components of the care process which scored lowest were the availability of laboratory services and safe blood supplies (50%), together with the availability of essential drugs, supplies and equipment (52.2%).

**Fig. 2:**
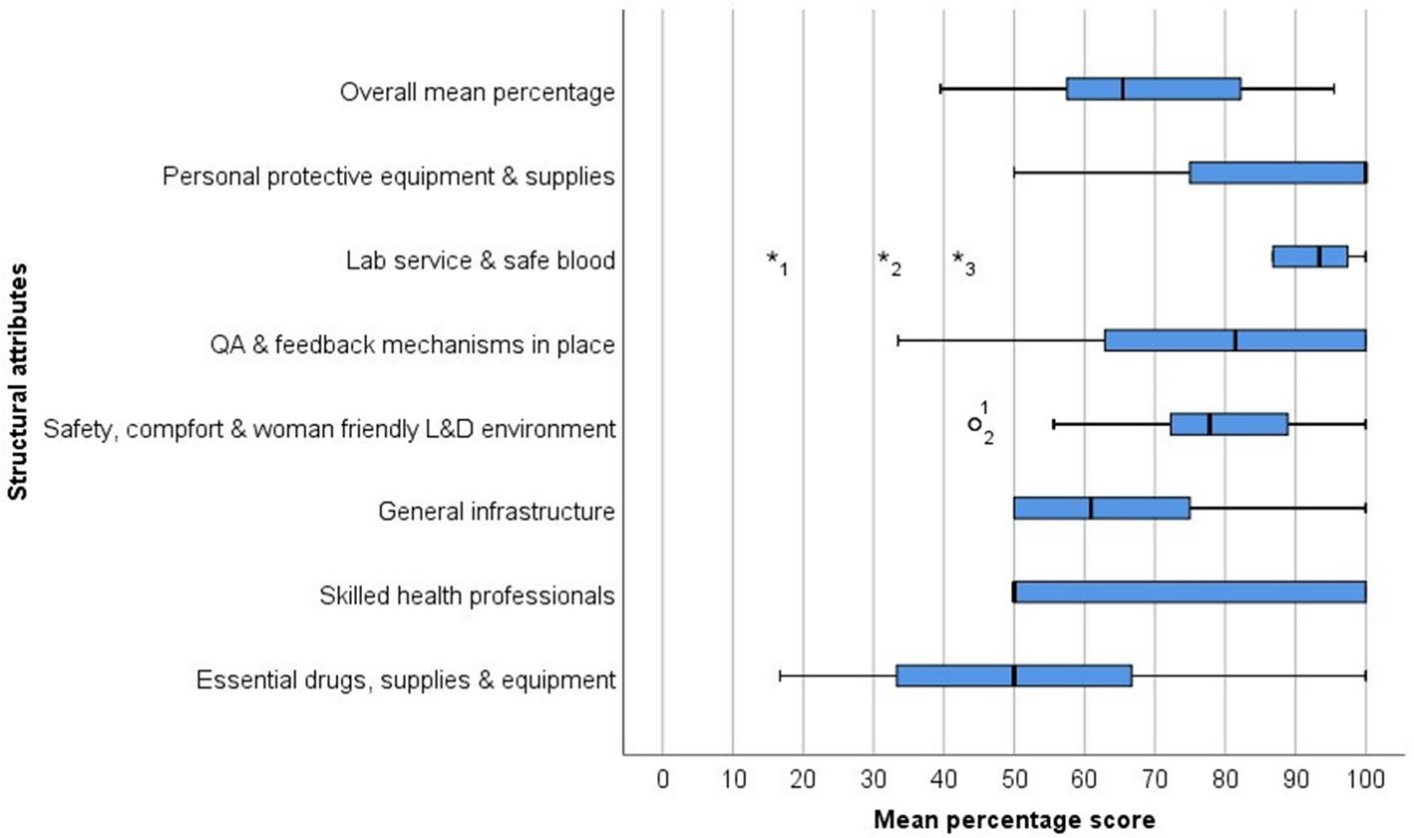
Distribution of median percentage scores of different structural attributes of quality L&D care at public hospitals in SNNPR, Ethiopia, 2016

The analysis also revealed that the median percentage scores varied among hospitals for many of the structural attributes. These included availability of skilled health professionals (median = 50.0; IQR = 50.0), QA and improvement activities with feedback systems (median = 81.5; IQR = 37.1), and essential drugs, supplies and equipment (median = 50.0; IQR = 33.4). In contrast, the median percentage score for laboratory service and safe blood supply was least variable among hospitals (median = 93.4; IQR = 10.6), followed by safety, comfort and woman friendly L&D environment (median = 77.8; IQR = 19.4). From plotting of outliers, two hospitals had lower median percentage scores for safety, comfort and woman friendly L&D environment domain compared to other hospitals. Similarly, three hospitals scored low for laboratory and safe blood services compared to other hospitals (Fig. 2).

## Discussion

Most hospitals had continuous electricity and water supply, which contrasts with previous studies [30, 41–43]. However, gaps were highlighted in the availability of telecommunication infrastructure, with less than half of the hospitals having had telephone service for internal communication, and only one hospital had the same in the compound for use by patients and their families. A study in Northern Ethiopia has reported that fewer facilities (12.5%) had a working phone or shortwave radio [41]. This compares to 100% of hospitals in Zambia and 88% in Uganda having functional communication systems [42]. However, this finding does not take into consideration the use of cell phones. This profound and technological change in telecommunications has not been updated within the Ministry of Health formal audit. However, cellphones are personal rather than institutional raising questions on payment and access. Functional communication systems facilitate communication among health facilities, contributing to improved referral networks for women with complications [42]. It also contributes to a positive experience of women with the care process, through improving responsiveness to their needs such as access to information and social and emotional support [44].

Availability of transport is important to ensure that women with complications are able to reach facilities that can provide appropriate care. A functional ambulance for emergency referral transportation was available in a great majority (90%) of the hospitals. This is much higher than the figures reported in previous studies in the region, including 65% [30], 59.4% [41], and 61% [42]. It is also slightly higher than 80% reported by the Ethiopia SARA 2016 [32]. This may be partly explained by a difference in the definition of the indicator. This study measured whether a functioning ambulance or other vehicle was stationed at the hospitals for emergency transport other studies have included an additional criterion of whether the vehicle had fuel available on the day of the survey [30], while others measured transport across a range of services including health centres and government, private and non-government managed hospitals. As financing and support systems are important features of operational environment affect facility performance, differences in supply chains and authorization are important. Government facilities depend on district and provincial administration for salary and material inputs [29].

Previous studies showed that women have limited opportunities to express opinions about their childbirth experience, or log complaints due to lack suggestion boxes or a log book [45]. Our study found that most hospitals had a suggestion box or log book in the labor ward for handling complaints. However, only half the suggestions were regularly evaluated, and documented. The lack of response may indicate a lack of accountability, and an undermining of women’s rights to seek justice for their mistreatment or malpractice [45].

The Ethiopian Ministry of Health requires that all health facilities have a written, up-to-date policy on triage and waiting times for emergency and non-emergency consultations and treatment for maternal and newborn healthcare [38]. However, one third of the hospitals did not have an emergency triage system for sick pregnant mothers who were not in labor. This is another gap that needs improvement.

Obstetric services must always be available, as an obstetric emergency can happen at any time [33]. One or more skilled attendants were present in all hospitals including weekends, for routine L&D care. Almost all hospitals had four or more fulltime midwives, though half the hospitals lacked a fulltime specialist in gynecology and obstetrics or a general practitioner. This compares favorably to 53.1% in Northern Ethiopia [41]. The recent SPA + Survey [30] and a facility survey in Uganda and Zambia [42] provided similar observations.

Yigzaw et al. [46] have identified lack of training among midwives as a barrier for provision of quality L&D care. Limited opportunities for refresher training on obstetric care may indicate less emphasis given to the importance of in-service training in improving the quality of L&D care [47]. Ongoing practice and periodic refresher training maintains obstetric management skills [48]. Skilled attendants were trained on management of L&D in the 12 months prior to this study in half of the hospitals, similar to Northern Ethiopia (56.3%) [41]. But it is higher than the finding reported in the Ethiopia SARA 2016 (33%) [32].

The required essential drugs, supplies and equipment should be kept in the L&D ward at all times to avoid unacceptable delay in providing the services [33]. This study showed that all essential drugs and equipment were available in only a quarter of the hospitals, and even fewer hospitals (15%) had all the supplies. However, oxytocin, an effective intervention to prevent postpartum hemorrhage was universally available, while magnesium sulphate, an effective intervention to manage pre/eclampsia, was missing in two hospitals.

Previous studies have also highlighted gaps in meeting the standards for essential drugs, supplies and equipment, though the degree of availability varies. For example, Getachew et al. [47] reported that 52.6% of the hospitals in Ethiopia (*N* = 19) had all the medicines and supplies needed for normal delivery, and 16% had magnesium sulfate. Centers for Disease Control and Prevention [42] also reported universal availability of oxytocin and magnesium sulfate in Zambian hospitals. Both studies documented higher availability of drugs than the present study (except for oxytocin) which may be partly explained by methodological and contextual differences between the studies. Hospitals from different managing authorities (government and missionary) across the country were included in the former study, while contextual difference and inclusion of different levels of facilities were in the latter study.

Shortage of HIV test kits and ARVs is a structural barrier faced by many hospitals. Official policy in Ethiopia is that all women with unknown HIV status should receive a rapid HIV test in maternity wards so that if diagnosed positive, ARV drugs can be given to the mother and baby in time to prevent vertical transmission [33]. Two hospitals lacked HIV test kits and ARV drugs for HIV positive mothers and for exposed babies. This situation in Ethiopia is similar to other African countries. In Zambia, HIV test kits were universally available and 50% of hospitals had ARVs drugs. In Uganda, 69% of the hospitals had ARV drugs [42]. To stop new vertical transmission of HIV, the coverage of Prevention of Maternal to Child Transmission (PMTCT) of HIV service should be above 85%. Yet, only 60.6% of HIV-positive Ethiopian women receive ART [49].

Notably, four in ten hospitals lacked towels for drying and wrapping newborns which highlights a critical gap in the hospitals’ capacity to promote thermoregulation in newborns. This is in agreement with previous work showing that this essential relatively inexpensive intervention had not been properly instigated. De Graft-Johnson et al. [48] identified this as the largest gap in the supply and Fisseha et al. [41] reported that the supply was available in 15.6% of the facilities.

Quality L&D care involves preventing avoidable infections thus the availability and accessibility of infection prevention IP equipment and supplies is essential in all facilities to enable health workers to adhere to the recommended hygiene practices [38]. Almost all hospitals had consumables for IP and adequate safety boxes and color-coded bins for waste segregation. Personal protective equipment was also fulfilled in most hospitals. This is better than a similar study which reported that the required personal protective items were available in two third of the facilities [41].

Offering quality L&D care also involves a welcoming and clean environment including access to a reliable supply of safe water and toilet facilities in maternity ward [33]. Almost all hospitals had well ventilated rooms, and the majority had tap water, sufficient space, a functional and clean toilet, and functional sinks with detergents. Availability of safe water supply is comparable with a study that reported 65.6% [41], but lower than the Centers for Disease Control and Prevention (CDC) [42] surveys which reported all hospitals in Uganda and Zambia having suitable supplies. This difference could be due to definitional criteria, with CDC using, regular water supply versus the specific definition used in this study, i.e., regular tap water supply.

The availability of toilet facility is favored by the Ethiopia SPA ( +)2014 Survey which reported an average of 74% of facilities having had a functioning latrine facility [30], but much higher than 34.4% reported in Northern Ethiopia [41]. Again, difference in the definition of the variable is the likely explanation for the deviation. The Northern Ethiopia study [41] measured the variable as availability of functional toilet and shower, while the current data did not specify the ‘shower’ element.

The capacity to conduct laboratory tests significantly enhances the quality of L&D care services. These require continuous supply of safe blood for emergency transfusions to treat hemorrhage [30]. However, critical gaps were identified with only 15% of hospitals having the range of tests needed for a mother in labor. This is consistent with other studies including the Ethiopia SPA ( +)2014 Survey [30], and those in Malawi with low scores for laboratory system [43].

The results of this study showed that 85% of the hospitals received blood from blood banks with safe storage practice, which is better than 75% in Ugandan hospitals [42]. This encouraging finding may reflect leadership commitment and efforts made to improve availability of safe blood and blood products in hospitals across Ethiopia. For instance, the existing blood banks have been reorganized by transferring the management of blood transfusion services from the Ethiopian Red Cross Society to government, the National Blood Transfusion Service. New blood banks are also constructed [49]. Due to lifesaving nature of the service, further efforts should be made to close the observed gap, even if small (15%). However, it is important to note that blood transfusion is an adverse outcome of childbirth related to problems of access and quality of haemorrhage prevention. Thus, structuring haemorrhage prevention should be given more emphasis.

All hospitals should display protocols and guidelines on MNHC for staff [33]. But only half of the hospitals had all essential guidelines and protocols in the L&D ward. The frequently missing guidelines for normal birth were management protocol on selected obstetrics topics (2010 version) and infection prevention protocol. Previous studies have also reported this poor performance [32, 43, 47, 50]. Accessibility to the best evidence determines successful implementation of evidence-based medicine [51], for example, the use of printed job aids that provide prompts to remind providers to perform specific tasks during intrapartum care is the most important predictor of quality of EmOC [52]. Thus, the result indicates another opportunity for QI.

Maternal and neonatal death review is important for improving the quality of MNHC services through systematic process of identifying factors associated with the deaths, generating recommendations to develop interventions against future similar deaths, and measuring improvement [33]. Almost all of the 20 hospitals conducted monthly maternal and neonatal death audits and implemented the recommendations. On the other hand, despite two thirds of the hospitals having a MNHC QI committee with a coordinator or focal person assigned, seven of the committees were not conducting regular meetings. This may be due to a lack of an organizational framework and standard procedures indicated in the EHAQ package [33]. Some hospitals were conducting death audits in the absence of a MNHC QI committee.

Inadequate performance of health facilities on various QA activities has also been observed in previous studies in Malawi [43] and Ethiopia [30]. For example, EPHI, FMoH of Ethiopia and ICF International [30] showed that half of all hospitals in Ethiopia had regular QA activities with observed documentation. Effective QA audit and feedback system in place can determine successful implementation of evidence-based practice [51]. There is a need to improve the ability to learn lesson from case reviews.

In summary, capacity gaps were observed in the hospitals to provide quality routine L&D care services with about two thirds of the required resources fulfilled overall. Only two hospitals had fulfilled almost all the standards, while one third of the hospitals had low readiness to deliver the service. Laboratory services and safe blood, and essential drugs, supplies and equipment were the areas with the largest gaps. These current findings are generally consistent with a recent study conducted in Northern Ethiopia on quality of delivery service where 65.62% of the facilities had good input quality [41], but much better than another study on quality of midwifery care in Amhara Region, Ethiopia, which reported 16.3% for availability of all essential drugs, and less than 10% for all essential equipment, all supplies, and all IP materials, each [46]. The present finding is also favored by other studies. For example, essential equipment for obstetric care was not always available in Kenyan hospitals [16] and health facilities in six sub-Saharan African countries including Ethiopia [48]. It is important to note that the results show just a single time point prevalence of the availability of the required inputs for the provision of quality care. This was the best possible assessment given the resources. Thus, they may not necessarily indicate a constant supply. Finally, it is worth remarking that evaluation of the structure alone is insufficient for a complete understanding of quality, because adequate processes may not follow even with a good structure.

### Limitations

The following limitations are acknowledged in this study. First, the results have limited generalizability to all public hospitals in the country, as the study was conducted only in SNNPR due to resource limitations. The quality of L&D care provided in the public hospitals of the region might differ from other regions. The use of locally adapted EHAQ standards could also compromise the external validity of the study. Representation of facilities from different managing authorities (government, private and non-government) is important as it implies differences in resource allocation, service requirements and working conditions, which can affect the quality of care they provide in different ways [2]. Thus, the results from this study may not even reflect the quality of L&D services provision in SNNPR, as it did not include public health centres and private and NGO owned facilities that provide skilled delivery care.

Second, composite scores are averages which can result in loss of important information [53]. The results were summarized using composite scores with apparent loss of item specific details. While acknowledging the limitation, composite measures have several advantages including: increased reliability than individual items [53] and giving the picture of both the whole and individual parts [10, 54]. This facilitates comparison and simplifies communication of performance on technical components of care to non-technical audiences [54].

Third, an extension of the above limitation relates to the use of unweighted scoring or equal weighting approach for the composite measures. It is acknowledged that assuming a linear relationship between all items and that they are equally important for the provision of quality L&D care might have impacted the results. The use of a weighted scoring was ruled out due to lack of evidence on the relative importance of the constructs of each quality component, though well-defined scientific evidence exists on the constructs, i.e., structural aspects of the EHAQ change package for L&D care [33]. Fourth, the availability of equipment, and essential drugs and supplies was based on point prevalence at the time of observation and does not necessarily indicate a constant supply, but this was the best possible assessment given the resources.

Fifth, the data captured on the availability of telephone service might not be valid as the facility audit checklist considered only fixed telephone service. However, due to the profound technological change in telecommunications in Ethiopia, the coverage of cell phones is wider than the landline telephone network. Sixth, information on refresher training was captured at hospital level through facility audit. The information might be less valid as it depended on the L&D case team leader’s verbal report which is subject to recall bias. Interviews with selected health professionals would rather provide a more valid measure of the indicator.

## Conclusions

Capacity gaps were observed in the hospitals to enable them provide quality routine L&D care services, with about two third of the required resources fulfilled overall. Only two hospitals had fulfilled almost all the standards, while one third of the hospitals had low readiness to provide quality routine L&D care. Laboratory services and safe blood, and essential drugs, supplies and equipment were the areas with the largest gaps. The following specific findings were evident.

Most hospitals had continuous electricity and water supply, and a functional ambulance service, but telecommunication infrastructure was inadequate. Women had opportunities to express their childbirth experience in most hospitals, but suggestions were regularly evaluated and documented in only half of the hospitals. Moreover, one third of the hospitals didn’t have emergency triage system for sick pregnant mothers who are not in labor.

Even though one or more skilled attendants were always present in all hospitals for routine L&D care, they received refresher training on obstetric care during 12 months prior to the study in only about half of the hospitals. Only a quarter of the hospitals had all essential drugs and equipment, and less than a quarter of the hospitals had all the required supplies. Notably, few hospitals lacked HIV test kits and ARV drugs limiting access to PMTCT of HIV services. Some hospitals lacked the capacity for thermoregulation of newborns due to lack of towels for drying and wrapping. Almost all hospitals had IP supplies and equipment to enable hygienic practices, and the majority had woman friendly L&D environment with sufficient space and a reliable supply of safe water and toilet facilities.

All the required laboratory tests were always available in only a few hospitals, whilst most hospitals were getting blood from blood bank with safe storage practice. Regarding adherence to QA practices, only half of the hospitals had all essential guidelines and protocols in the L&D ward, highlighting that the management protocol on selected obstetrics topics (2010 version) and an infection prevention protocol were frequently missing. Furthermore, some hospitals were conducting monthly maternal and neonatal death audits and implementing recommendations in the absence of a supportive organizational framework, i.e., a MNHC QI committee, with a focal person assigned, that meets regularly.

### Recommendations

The results suggest the need to ensure that all public hospitals in SNNPR meet the required structure to enable the provision of quality routine L&D care. This is with an emphasis on laboratory services and safe blood for emergency transfusion, and essential drugs, supplies and equipment. Hospitals should establish responsive telecommunication and emergency triage system for mothers. Improving access to safe water supply and toilet facility needs an urgent attention to ensure that the L&D environment is woman friendly.

There is a need for routine monitoring of the adequacy of essential drugs, supplies and equipment. Particular attention should be given to HIV test kits and ARV drugs. Hospitals also need to have adequate towels for drying and wrapping newborns. The capacity of hospitals should also be strengthened to conduct basic laboratory tests for women, giving emphasis to liver and renal function tests, serum protein and albumin tests, CD4 count or HIV plasma viral load tests, and HIV tests.

All essential L&D care guidelines and protocols for normal delivery should be made available in all hospitals, with emphasis to the management protocol on selected obstetrics topics 2010 version and IP protocol. Hospitals should encourage women to express opinions about their childbirth experience and take appropriate remedial actions based on the feedback. Hospitals should also conduct regular maternal and neonatal death audits in compliance with the standard procedures and supportive organizational framework showed in the EHAQ package [33]. A strong leadership is also important to warrant completion of the audit cycle.

Supplementing with qualitative data through health worker interviews in a similar future research would be important in explaining some of the findings. Representation of facilities from different managing authorities (government, private and non-government) is also important as it can have a direct or indirect effect on the quality of care they provide.

## Data Availability

The dataset used and analysed during the current study is available from the corresponding author on reasonable request.

## Abbreviations

ART: Anti-retroviral therapy
ARV: Anti-retroviral
BEmONC: Basic emergency obstetric and newborn care
CSF: Cerebrospinal fluid
EHAQ: Ethiopian hospital alliance for quality
EmOC: Emergency obstetric care
EPHI: Ethiopian Public Health Institute
FMoH: Federal Ministry of Health
GP: General Practitioner
HO: Health Officer
IESO: Integrated Emergency Surgical Officer
IP: Infection prevention
L&D: Labor and Delivery
MNHC: Maternal and neonatal health care
PMTCT: Prevention of maternal to child transmission
QA: Quality assurance
QI: Quality improvement
SNNPR: Southern Nations Nationalities and People’s Region
SPA: Service provision assessment
SSA: Sub-Saharan Africa
TTC: Tetracycline
